# Effects of increasing cognitive demands through expanding movement options on biomechanics during changes of direction in female football players

**DOI:** 10.1038/s41598-025-25069-2

**Published:** 2025-10-23

**Authors:** Clara Ebner, Urs Granacher, Dominic Gehring

**Affiliations:** Department of Sport and Sport Science, Exercise and Human Movement Science, University of Freiburg, Sandfangweg 4, 79102 Freiburg, Germany

**Keywords:** Anterior cruciate ligament, Cognition, Female football, Kinematics, Musculoskeletal system, Psychology

## Abstract

Anterior cruciate ligament injuries often occur during changes-of-direction (CODs), particularly when combined with cognitively demanding decision-making tasks. This study investigated the effects of increasing movement options during CODs in response to a real opponent on whole-body biomechanics in female football players. Twenty-nine female football players (15 with high and 14 with low expertise) performed 90° CODs in response to a real opponents’ action under four conditions: anticipated with one option (ANT-1), unanticipated with two (UNANT-2), three (UNANT-3) or four (UNANT-4) movement options. Three-dimensional motion analysis captured whole-body biomechanics at initial contact and during weight acceptance. Continuous biomechanical data were analyzed using a statistical parametric mapping approach. No significant condition effects were observed for peak knee mechanics. However, at initial contact the pelvis was significantly less tilted and rotated towards the running direction in the UNANT-4 condition than in ANT-1. The hip was significantly more abducted and internally rotated in all unanticipated CODs. Furthermore, trunk rotation to the cutting leg was reduced in all unanticipated conditions compared to ANT-1. No significant differences were found between expertise groups. Increasing cognitive demands in a simulated match-play scenario primarily influenced proximal segment biomechanics during CODs in female football players. The authors therefore recommend integrating whole-body control and cognitively demanding stimuli into testing and injury prevention strategies.

## Introduction

Anterior cruciate ligament (ACL) injuries are a major concern in sports due to their high prevalence rates and long-term health consequences for athletes^1^. In female football, approximately 0.7 ACL injuries, including complete and partial ruptures, per team and season can be expected, which corresponds to an injury risk 4 to 6 times higher than in male football players^2^. Notably, the ACL injury risk is comparable across performance levels in female football, with similar incidence rates in amateur-level and professional players^3^. ACL ruptures are reported to be the most burdensome injury in female football, with a median of 292 days from injury to return to play^1^.

Understanding the underlying biomechanical risk factors is crucial for the design and implementation of effective injury prevention strategies^4^, such as neuromuscular training. The majority of ACL injuries in female football are non-contact (54%) or indirect contact (34%) injuries^5^. More specifically, these injuries occur without direct player or opponent contact to the injured knee. These injuries predominantly arise in complex defensive playing situations, like pressing and tackling, which require rapid deceleration and change-of-direction (COD) maneuvers to follow the offensive player^5,6^.

Biomechanical analyses of ACL injury events provide valuable insights into movement patterns associated with an elevated injury risk. A video analysis of 29 ACL injuries in female football identified knee valgus alignment in 88% of the ACL injury events accompanied by an abducted hip and increased hip internal rotation from the initial contact (IC) to the moment of injury occurrence^5^. Lucarno et al. (2021) further observed that players often show lateral trunk flexion to the injured limb and trunk rotation toward the uninjured limb, i.e., in the intended running direction^5^. These findings underscore the importance of considering whole-body kinematics when investigating ACL injury paradigms with the goal to develop targeted prevention strategies.

In recent years, laboratory-based ACL injury risk assessment approaches have expanded beyond focusing on biomechanical aspects by including cognitive aspects, especially when addressing non-contact ACL injuries. Notably, ACL injuries often occur during complex match-play situations, where athletes must react rapidly to an opponent’s action^7^. The shift of attentional focus from the player’s own COD action towards an external stimulus introduces a situation, in which cognitive-motor interference (CMI) may occur^8^. This interference can impair players’ temporo-spatial perception and it may compromise movement control, as cognitive resources are limited and thus divided between decision-making and motor execution^7^. Additionally, deceiving actions, such as feints, further increase the cognitive demands by requiring defenders to inhibit an intended, pre-planned or even an already initiated motor response^7,9^. The inherent time constraints of these match-play situations challenge appropriate feed-forward control strategies, which are essential to stabilize the whole body and particularly the knee joint^10^. Given that ACL injuries typically occur within 50 ms after initial ground contact, reactive mechanisms, such as muscle reflexes, are often insufficient to mitigate the underlying injury risk^9,11^. This highlights the crucial role of executive functions, particularly inhibitory control and cognitive flexibility, for rapid and accurate decision-making under time pressure^10^. Athletes experienced in open-skill sports such as football, basketball and handball may be less affected by cognitive-motor demands due to constant exposure to comparable situations during training or match play. Empirical evidence indicates that executive functions improve progressively with higher levels of sport expertise^12^. Thus, understanding the impact of elevated cognitive demands during complex match-play situations on whole-body kinematics appears crucial to better comprehend the mechanisms underlying ACL injuries^7,13,14^.

Hughes and Dai (2021) proposed a hypothetical model illustrating how cognitive demands may affect motor control in highly dynamic match-play situations^14^. The model emphasizes decision-making processes by distinguishing between anticipated (pre-planned) CODs and unanticipated movements, with the latter requiring reactive adjustments in response to external stimuli, such as an opponent’s action^14^. Accordingly, decision-making processes are mainly affected by the number and complexity of movement options, as well as the available time to react (ATR)^14^.

Emerging evidence from laboratory-based studies indicate that cognitive demands, being operationalized as unanticipated versus anticipated CODs, modulate knee joint mechanics and whole-body kinematics^13,15^. More specifically, higher cognitive demands have been associated with increased knee abduction moments^13,15^, greater lateral trunk flexion to the cutting leg and rotation to the intended running direction^16,17^, as well as increased hip abduction and internal rotation during weight acceptance^18,19^. Several research groups have investigated the influence of ATR, defined as the time from stimulus to IC, on biomechanical parameters associated with ACL injury risk^16,20^. The authors have found higher knee abduction moments and angles as well as increased lateral trunk flexion and rotation in CODs with an ATR of 600 ms compared to 700 ms and 850 ms^16,20^. Findings revealed that an ATR of around 600 ms is required to differentiate between anticipated and unanticipated CODs, following an approach run at a speed of 4–5 m/s^16,20,21^.

Previous research primarily relied on artificial stimuli, such as light signals or arrows, to create unanticipated conditions with 2 (e.g., left or right) or 3 (e.g., left, right or straight) possible movement options^14,21^. However, only few studies have examined the effects of decision-making using ecologically valid stimuli to enhance task complexity and better simulate match-play scenarios^14,18,22^. For instance, Lee et al. (2013)compared the effects of ecologically valid stimuli (i.e., 1 vs. 2 video-animated opponents) versus an artificial stimulus (i.e., arrow) on knee mechanics and trunk kinematics in football players of low versus high performance and training caliber while performing COD tasks with 2 options (right/left)^17^. Higher knee abduction moments were found in unanticipated versus anticipated tasks, with the highest moments and most unfavorable trunk kinematics in arrow-induced CODs compared to the condition with video-animated opponents^17^. Furthermore, only the condition with video-animated opponents was able to discriminate between performance levels, with low-level players demonstrating a less favorable COD strategy^17^. In summary, ecologically valid stimuli seem to increase cognitive demands in match-play situations, especially in players with limited sport-specific expertise and potentially less developed visual-perceptual skills^17^.

Despite increasing interest in the role of cognitive demands on ACL injury risk, no studies are currently available that have examined the effects of cognitive demands, by systematically increasing the number of movement options in a complex match-play scenario. Finally, the available research has primarily focused on knee and hip biomechanics, despite the multi-segmental nature of ACL injuries involving the trunk, pelvis, and foot^21^.

Therefore, the primary aim of this study was to investigate the effects of increasing cognitive demands on whole-body biomechanics associated with an ACL injury risk during COD task performance in female football players. Cognitive demands were systematically increased across 4 levels by varying the number of movement options in a complex football-specific laboratory setting. Specifically, participants were required to rapidly perceive and interpret external cues, inhibit pre-planned motor responses if necessary, and select an appropriate movement strategy under time pressure. A secondary aim was to examine whether football-specific expertise moderates the influence of cognitive demands on biomechanical responses by comparing female football players of high versus low training and performance caliber.

Based on the relevant literature^17,18,22^, it was hypothesized that the gradual increase of cognitive demands during COD task performance may amplify knee joint mechanics associated with ACL injuries (e.g., knee abduction angles and moments), as well as whole-body kinematics such as trunk and hip alignment. Furthermore, it was hypothesized that higher football-specific expertise may reduce the influence of increasing cognitive demands on ACL injury-associated biomechanics^17^.

## Methods

Using a within-subject repeated-measures design, this study examined the effects of 4 levels of cognitive demands on biomechanics during COD task performance. The study protocol was preregistered on the Open Science Framework and is available at [10.17605/OSF.IO/4Z5R8].

### Participants

Sample size estimation for the primary research question was based on the effect sizes for knee abduction moments and trunk kinematics (f = 0.27–0.29) of a study with related study design^17^. The a priori power analysis^23^ revealed a minimum required sample size of 21 participants to achieve 80% statistical power at an alpha level of 0.05. To account for potential data loss and facilitate analyses addressing the secondary objective, a total sample size of 30 participants was targeted.

Accordingly, 30 female athletes volunteered to participate in this study. One participant had to be excluded due to an adverse event during an approach run, resulting in a final sample of 29 participants. Participants completed a questionnaire that included questions on anthropometrics, their current health status assessed via the Physical Activity Readiness Questionnaire (PAR-Q), previous lower limb injuries and their sporting background, including football-expertise.

The participants were categorized into 2 groups according to their training and performance caliber. The high expertise group (HE) included 15 players aged 22.9 ± 3.8 years (body height = 168.5 ± 4.7 cm, mass = 63.9 ± 5.1 kg) and the low expertise group (LE) included 14 participants aged 22.5 ± 1.8 years (body height = 168.6 ± 5.6 cm, mass = 63.6 ± 5.6 kg).

HE participants competed in the 1 st to 4th German football leagues, with an average of 14.7 ± 3.8 years of club-level playing experience and 8.1 ± 5.1 h of play per week. In contrast, LE participants had limited football experience, having completed only 1 year of a university football course and averaging 0.5 ± 0.7 playing hours per week. However, 6 of the LE participants were engaged in other team sports such as basketball or volleyball.

All participants were free of lower limb injuries within the past 3 months prior to the start of the study. Previous ACL injuries were not an exclusion criterion if they had occurred or were surgically treated at least 24 months prior to study participation. Three participants had previously suffered an ACL injury (HE = 2; NE = 1), two of whom had been treated surgically.

Prior to testing, all participants were informed about potential risks and provided written informed consent. The study was conducted in accordance with the latest version of the Declaration of Helsinki, and the protocol was approved by the local ethics committee of University of Freiburg, Germany (approval number 24–1142-S2).

### Experimental setup and conditions

Approximately 60% of ACL injuries in female football players occur in defensive playing situations like pressing^5^. Therefore, the experimental setup was designed to simulate a match-like defensive scenario. Participants performed COD tasks, in which they adopted the role of a defender against a real opponent in ball possession. Each movement task included a submaximal approach run of 5 m at 4.0 ± 0.3 m/s, followed by a 90° COD to the left or the right in response to the opponent who determined the direction of the participant by kicking a ball to one side. To ensure reliable and consistent testing conditions, two experienced male footballers competing at regional level were selected as opponents and were equally assigned to participants from the HE and LE groups.

**Fig. 1:**
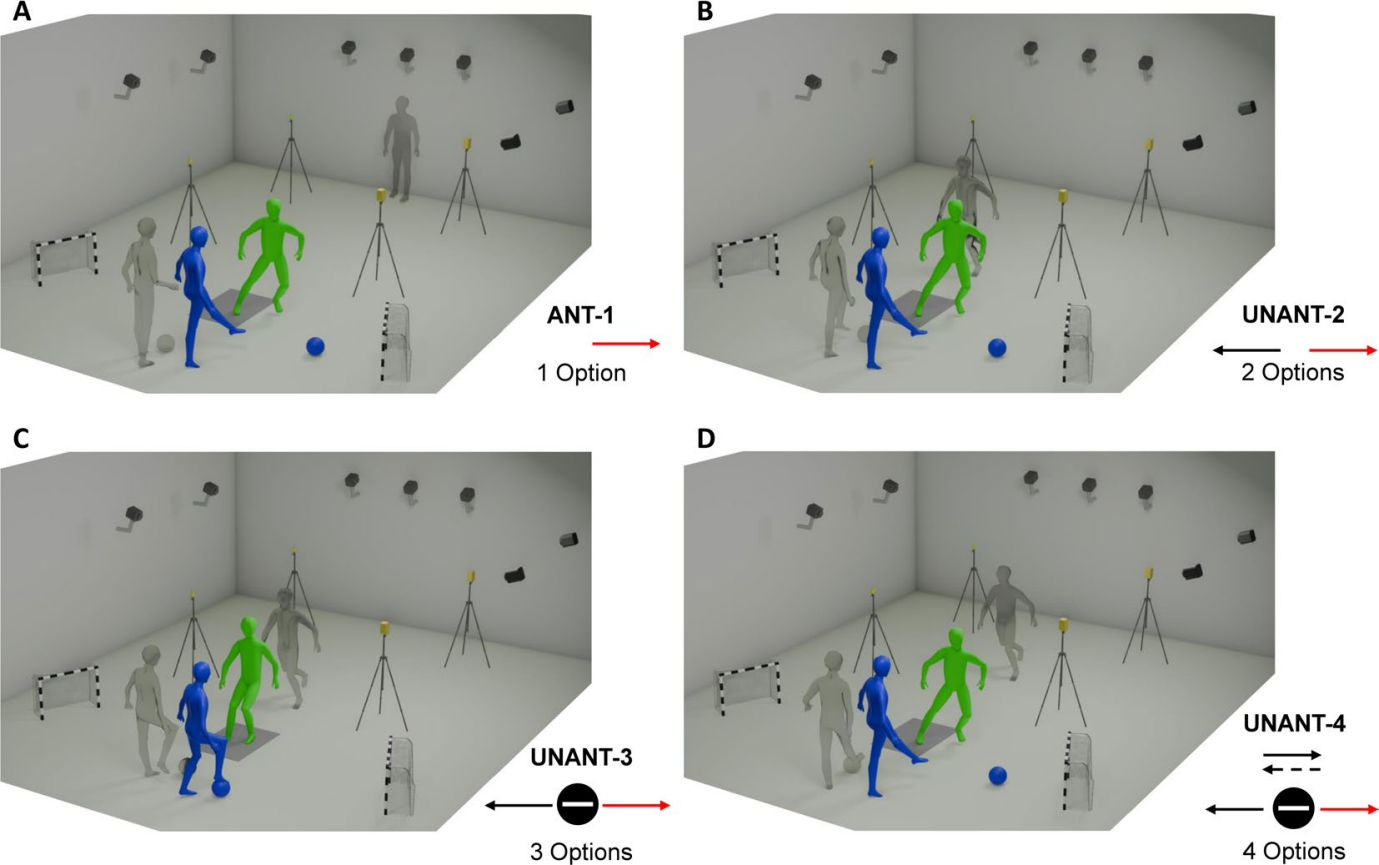
Exemplified illustration of one movement option for each experimental condition. Further movement options can be found in the description of the conditions in the text. The blue figures represent the opponent, the green figures the participant during COD task performance. The red arrows indicate the analyzed movement option. (**A**) ANT-1: the grey figures represent the starting positions of the subject and the opponent, with the latter pointing in the desired cutting direction. (**B**) UNANT-2: the grey figures represent the time of the stimulus provided by the opponent by kicking the ball. (**C**) UNANT-3: the grey figures represent the time of the stimulus provided by the opponent by stopping the ball. (**D**) UNANT-4: the grey figures represent the time of the feint, initiated by the opponent shortly before he kicked the ball to the other side. *Illustration created by Roland Blechschmied*,* used with permission.*

The participants performed CODs under experimental conditions with increasing cognitive demands, presented in block-randomized order for each participant. In the anticipated (ANT-1) condition (Fig. 1A), participants performed a COD to the left or the right, which was indicated by a hand signal of the opponent prior to the approach run and therefore resulted in 1 option for the participant. The opponent then performed an inside kick to the right or the left at a standardized time point during the participants’ approach run. In the unanticipated condition with 2 options (UNANT-2, Fig. 1B), participants initiated the approach run without prior knowledge of the cutting direction, requiring them to react rapidly as the opponent kicked the ball to either side at the same standardized time point as in the ANT-1 condition. The unanticipated condition with 3 options (UNANT-3, Fig. 1C) further increased the cognitive demand by introducing an additional third movement option. Here, the opponent either passed the ball left or right or stopped it by placing the foot on top of the ball, requiring the participant to decelerate and stop quickly in front of the ball. The unanticipated condition with 4 options (UNANT-4, Fig. 1D) provided the highest number and complexity of options by containing all previous movement options along with a deceptive feint in several trials. Performing the feint, the opponent initially moved the foot toward the ball, indicating a pass to one direction before quickly switching the supporting leg and kicking the ball to the opposite side. To ensure temporal comparability across conditions, the feint was initiated slightly earlier, ensuring that ball kicking occurred at a timepoint comparable to the other conditions. In accordance with recent evidence^21^, an ATR of 300–600 ms was adopted. The visual cue for the opponent to kick the ball was the moment the participant passed the second timing gate, placed at a distance of 1.5 m from the center of the force plate. The selected approach run speed of 4 m/s resulted in an ATR of approximately 375 ms.

Participants performed CODs on a standard laboratory floor wearing neutral indoor football shoes (Mundial Goal, Adidas, Herzogenaurach, Germany). Prior to data collection, participants completed a standardized warm-up protocol to prepare for fast, dynamic movements. Subsequently, participants performed at least 3 familiarization trials in each experimental condition.

To prevent modification in stride length or movement patterns, participants were not instructed to target the force plate during COD execution. However, complete foot contact of the cutting leg on the force plate was required for valid trials. During familiarization, the preferred cutting leg was determined, and only CODs of this leg were taken for further data analysis. For clarity, only the 90° CODs with the preferred leg were analyzed. The other movement options, i.e., CODs to the non-preferred side, CODs after the feint and the stopping maneuvers, served solely to increase cognitive demands but were not included in the final analysis.

Each participant performed at least 14 trials per condition, 7 of which were CODs with the preferred cutting leg. If fewer than 5 valid trials were recorded due to missing the force plate or deviating from the desired approach speed, further trials were performed until meeting the required number of 5 valid trials.

### Data collection and analysis

The approach run speed was measured by two timing gates (Witty Gate, Microgate, Mahopac, NY, USA), placed at a distance of 3.5 m and 1.5 m from the center of the force plate.

Three-dimensional motion data of the participant, the opponent and the ball were collected at 200 Hz using a marker-based motion analyses system with 12 cameras (Vicon Motion Systems Ltd., Oxford, Great Britain). Ground reaction forces (GRF) were recorded at 1000 Hz using a ground-embedded force plate measuring 0.9 m x 0.6 m (AMTI BP600900, Watertown, MA USA). Motion capture data and GRF were synchronized (Vicon D-Link) to allow inverse dynamic calculations.

A customized marker set was used to analyze players’ trunk, lower limbs and foot kinematics, and to track the movement initiation of the ball and opponent. Based on a previously established marker set^16^, 37 markers were placed on the participant’s head (4 markers), trunk (suprasternal notch, xiphoid process, T6 vertebra), pelvis (anterior and posterior superior iliac spines), legs (lateral thigh and shank, medial and lateral epicondyle of the knee, tuberositas tibiae, medial and lateral malleolus) and shoe (3-marker clusters on the forefoot and rearfoot). To track the ball movement, a 3-marker cluster was attached to the ball. Additionally, the opponent was equipped with 18 markers, distributed across specific landmarks on the whole body and the shoes. To ensure reliability for marker placement, the same experienced researcher placed all markers across participants.

Marker trajectories were pre-processed with regards to labelling and gap-filling using the built-in Woltring and Rigid Body Fill tools in Vicon Nexus. For the Woltring interpolation, the maximum gap length was set to five frames. Rigid Body Fill was solely used for segments with at least four markers, i.e., pelvis and head segment, with a maximum gap length of 25 frames. Of note, due to our setup with 12 cameras focusing on a very defined field of view, gap filling was rarely necessary within the temporal and spatial region of interest. Marker trajectories and GRF signals were finally filtered with a low-pass Butterworth filter (4th order, 20 Hz cut-off frequency) in Vicon Nexus. A static calibration trial was conducted with the participant standing in a predefined neutral position within a foot calibration rig to determine segment length and joint centers. Ankle and knee joint centers were defined as the midpoint between the medial and lateral malleoli and medial and lateral epicondyles, respectively^16,24^. Hip joint centers were functionally determined using a standardized dynamic movement protocol (“star-arc movement”)^25^.

Segment coordinate systems and calculations of joint angles and joint moments were established using a custom-written script in BodyBuilder (Vicon Motion Systems Ltd., Oxford, UK). Briefly, vertical axes for the thigh and shank were defined from distal to proximal joint centers, with the mediolateral axes being defined through the medial and lateral epicondyle and malleolus markers, and the anteroposterior axes resulting from the cross product. Pelvis and trunk segment axes were defined to match with previous publications^16,26^. Joint angles were computed using a YXZ Euler rotation sequence. Knee joint rotations were defined as flexion-extension around the Y-axis, adduction-abduction around the subsequently X’-axis, and internal-external rotation around the Z’’-axis^16^. External knee joint moments were then calculated using a standard inverse dynamics approach and normalized to each participant’s body mass. The IC was set to the frame in which vertical GRF exceeded a value of 20 N. All further processing was performed using custom scripts in Matlab (R2022b, The MathWorks Inc.).

The discrete biomechanical variables were selected based on their association with the ACL injury mechanism^5,13^. At IC, knee flexion and foot progression angles as well as pelvis, hip and trunk angles in the frontal and transverse plane were extracted, reflecting preparatory movements for initiating the COD^16^. Furthermore, the peak knee abduction angle and moment were extracted during weight acceptance (WA), as this early phase of stance can be considered relevant for ACL injuries^9^. WA was defined as the time from IC to maximum knee flexion^27^. A detailed description of the joint angles and moment directions will be provided in the results section.

The control parameters approach speed and ATR were analyzed to assess their consistency within and between subjects. As it was uncertain whether participants primarily reacted to opponent or ball movements, both measures were reported. ATR_Opp_ was defined as the time from initial movement of the opponent’s kicking foot until IC, while ATR_Ball_ was defined as the time from initial ball movement until IC.

### Statistical analyses

Statistical analyses of discrete data were computed in R software (version 2023.12.0 + 369). For each participant and experimental condition, the dependent biomechanical variables were averaged across 5 trials. Normal distribution of data was confirmed using the Shapiro-Wilk test and by visual inspection of Q-Q-Plots. Homogeneity of variance was confirmed using the Levene’s test. A mixed 4 × 2 ANOVA was used to examine the effects of the within-subject factor *condition* (4 levels: 1, 2, 3, 4 options) and the between-subject-factor *expertise* (2 levels: high, low). Statistical significance was set at *p* < 0.05, and significant main group and condition effects and interactions thereof were followed up with paired t-tests using Bonferroni correction. Effect sizes of the ANOVA analyses were reported as partial eta squared (η_p_^2^) and can be interpreted as small (< 0.06), medium (0.06–0.14) and large (> 0.14) effects^28^. Cohen’s d was calculated to assess the effect sizes of paired t-tests and can be interpreted as small (< 0.5), medium (0.5–0.8) and large (> 0.8) effects^28^.

Additionally, a statistical parametric mapping (SPM) approach was performed to analyze continuous biomechanical data using the spm1d package in MATLAB (R2022b, The MathWorks Inc.). The WA phase was used for analyses. A one-way repeated-measures ANOVA was performed to assess differences in biomechanical variables between the 4 experimental conditions. If ANOVA tests reached significance, Bonferroni adjusted paired t-tests were calculated.

To assess the reliability of the experimental conditions, intraclass correlation coefficients (ICCs) and their 95% confident intervals (CI) were calculated for the control parameters approach speed, ATR_Ball_ and ATR_Opp_ using a two-way random effects model (ICC(2, k))^29^. ICC estimates were interpreted as poor (< 0.5), moderate (0.5–0.75), good (0.75–0.9), and excellent (> 0.9) reliability^29^.

## Results

### Control parameters

The approach speed remained within the required range of 4.0 ± 0.3 m/s (Table 1), with no significant effects of condition (*p* = 0.117, η_p_^2^ = 0.07) or expertise (*p* = 0.181, η_p_^2^ = 0.07). Similarly, both time-to-react variables, i.e., ATR_Opp_ and ATR_Ball_, did not differ between conditions (*p* = 0.703, η_p_^2^ = 0.02 and *p* = 0.073, η_p_^2^ = 0.08, respectively) or between expertise levels (*p* = 0.21, η_p_^2^ = 0.06 and *p* = 0.144, η_p_^2^ = 0.08, respectively) (Table 1). All control parameters showed good between-condition reliability with ICCs of 0.89 [95% CI: 0.82, 0.94] for approach speed, 0.76 [95% CI: 0.63, 0.89] for ATR_Opp_ and 0.79 [95% CI: 0.68, 0.86] for ATR_Ball_.

**Table 1:** Means ± SDs of the control parameters approach speed, ATR_Opp_ and ATR_Ball_ for the 4 experimental conditions.

	Expertise	ANT-1	UNANT-2	UNANT-3	UNANT-4
Approach speed [m/s]	HE	3.99 ± 0.21	4.05 ± 0.24	4.00 ± 0.21	4.02 ± 0.21
	LE	3.90 ± 0.12	3.92 ± 0.18	3.90 ± 0.21	3.94 ± 0.19
ATR_Opp_ [s]	HE	0.70 ± 0.06	0.71 ± 0.07	0.72 ± 0.07	0.70 ± 0.06
	LE	0.74 ± 0.09	0.74 ± 0.09	0.75 ± 0.11	0.74 ± 0.09
ATR_Ball_ [s]	HE	0.34 ± 0.07	0.36 ± 0.07	0.37 ± 0.08	0.36 ± 0.05
	LE	0.39 ± 0.10	0.41 ± 0.10	0.40 ± 0.11	0.40 ± 0.10

*ANT = anticipated*,* ATR = available time to react*,* HE = high expertise*,* LE = low expertise*,* UNANT = unanticipated*

### Discrete biomechanical variables

No significant main condition effects were observed for any of the knee joint related discrete variables, including peak knee abduction moment, peak knee abduction angle, and knee flexion angle at IC (see Table 2).

**Table 2:** Knee kinetics and kinematics during change-of-direction task performance: means ± SDs and statistics.

	Expertise	ANT-1	UNANT-2	UNANT-3	UNANT-4	Condition effect (*p*-value, η_*p*_^2^)	Expertise effect (*p*-value, η_*p*_^2^)	Condition*expertise effect (*p*-value, η_*p*_^2^)
Peak knee abduction moment [Nm/kg]	HE	0.88 ± 0.42	0.90 ± 0.33	0.86 ± 0.33	0.96 ± 0.38	0.075 (0.081)	0.716 (0.005)	0.180 (0.058)
	LE	0.78 ± 0.29	0.83 ± 0.33	0.91 ± 0.37	0.90 ± 0.38			
Peak knee abduction angle [°]	HE	9.5 ± 5.0	10.5 ± 5.5	9.5 ± 5.0	9.8 ± 4.9	0.516 (0.028)	0.729 (0.005)	0.081 (0.079)
	LE	10.6 ± 3.8	10.0 ± 3.8	10.2 ± 3.6	10.7 ± 3.6			
Knee flexion angle at IC [°]	HE	28.2 ± 6.8	29.9 ± 7.3	30.1 ± 9.3	29.5 ± 6.7	0.625 (0.021)	0.146 (0.077)	0.817 (0.011)
	LE	25.7 ± 6.9	25.6 ± 7.5	26.4 ± 6.4	25.4 ± 6.9			

*ANT = anticipated*,* IC = initial contact*,* HE = high expertise*,* LE = low expertise*,* UNANT = unanticipated*

In contrast, significant condition effects were observed for proximal joint and segment kinematics. Regarding hip and pelvis kinematics at IC (Table 3), significant main condition effects were found for hip rotation (*p* = 0.034, η_p_^2^ = 0.101), frontal pelvis tilt (*p* = 0.004, η_p_^2^ = 0.149) and pelvis rotation (*p* = 0.012, η_p_^2^ = 0.125). Post-hoc analyses revealed that the pelvis was significantly less tilted and rotated towards the running direction in the UNANT-4 condition, i.e., in unanticipated CODs with the highest cognitive demand, than in ANT-1 (*p* = 0.006, d = −0.686 and *p* = 0.041, d = 0.543, respectively). Post-hoc analyses for hip rotation did not reach the level of statistical significance. Lateral trunk flexion at IC was not significantly affected by the condition (Table 3). However, there was a significant condition effect on trunk rotation at IC (*p* < 0.001, ηp^2^ = 0.249). Post-hoc analyses revealed that the trunk was significantly more rotated to the cutting leg in the ANT-1 condition compared to UNANT-2 (*p* = 0.023, d = −0.585), UNANT-3 (*p* = 0.002, d = −0.745) and UNANT-4 (*p* = 0.007, d = −0.669). No significant condition effects were found for foot progression angle at IC (Table 3).

**Table 3:** Kinematics of the pelvis, hip, trunk and foot at initial contact (IC) during change-of-direction task performance: means ± SDs and statistics.

	Expertise	ANT-1	UNANT-2	UNANT-3	UNANT-4	Condition effect (*p*-value, η_*p*_^2^)	Expertise Effect (*p*-value, η_*p*_^2^)	Condition*expertise effect (*p*-value, η_*p*_^2^)
Frontal pelvis tilt [°] (- = towards running direction, i.e., iliac crest higher on the side of the cutting leg)	HE	−12.4 ± 4.6	−11.7 ± 5.2	−11.2 ± 4.0	−11.2 ± 5.0	**0.004 (0.149)**	0.086 (0.105)	0.770 (0.014)
	LE	−9.9 ± 5.4	−8.3 ± 5.6	−7.9 ± 4.9	−7.7 ± 5.4			
Pelvis rotation [°] (+ = towards running direction)	HE	22.8 ± 9.2	22.1 ± 7.3	21.6 ± 7.4	20.9 ± 6.9	**0.012 (0.125)**	0.457 (0.021)	0.291 (0.045)
	LE	23.3 ± 12.3	18.9 ± 11.3	18.8 ± 8.4	17.2 ± 10.8			
Hip abduction/adduction [°] (+ = abduction)	HE	8.3 ± 5.5	9.6 ± 4.7	9.6 ± 4.9	9.6 ± 5.6	0.405 (0.035)	0.689 (0.006)	0.652 (0.020)
	LE	9.8 ± 4.3	10.0 ± 5.4	10.2 ± 3.8	9.9 ± 5.0			
Hip rotation [°] (+ = internal rotation)	HE	2.6 ± 11.1	4.5 ± 11.5	5.8 ± 11.6	6.0 ± 12.7	**0.034 (0.101)**	0.373 (0.030)	0.716 (0.016)
	LE	7.1 ± 8.6	7.9 ± 9.8	8.3 ± 8.6	8.9 ± 7.4			
Lateral trunk flexion [°] (+ = to the cutting leg)	HE	16.0 ± 5.4	15.8 ± 4.3	15.5 ± 4.5	16.5 ± 5.2	0.541 (0.026)	0.069 (0.117)	0.974 (0.003)
	LE	12.6 ± 6.5	12.1 ± 5.9	11.8 ± 5.7	12.5 ± 7.1			
Trunk rotation [°] (- = to the cutting leg)	HE	−17.3 ± 7.7	−13.9 ± 5.7	−13.6 ± 6.2	−14.1 ± 6.9	**< 0.001 (0.249)**	0.181 (0.065)	0.561 (0.025)
	LE	−13.3 ± 10.0	−11.7 ± 7.6	−9.7 ± 6.3	−10.3 ± 7.3			
Foot progression [°] (+ = internal rotation)	HE	11.0 ± 10.5	11.9 ± 9.2	10.7 ± 10.5	10.3 ± 10.9	0.312 (0.043)	0.169 (0.078)	0.478 (0.030)
	LE	17.8 ± 9.7	15.2 ± 8.3	13.9 ± 9.1	14.6 ± 6.5			

*IC = initial contact*,* HE = high expertise*,* LE = low expertise*

No significant main group effects or condition-by-group interaction effects were observed for none of the selected biomechanical variables.

### Continuous biomechanical variables

**Fig. 2:**
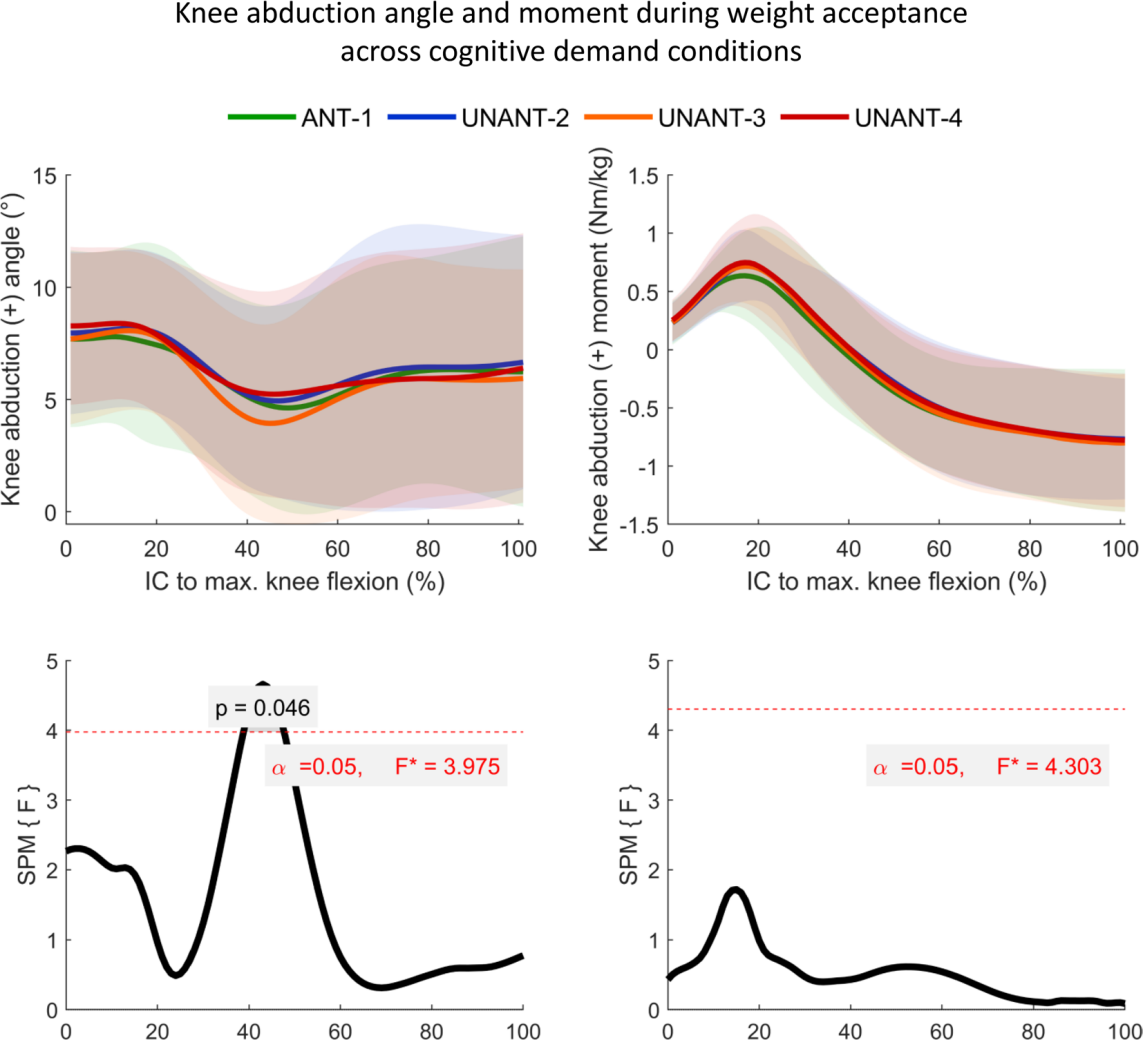
Top row: Means (± SD shaded areas) of knee abduction angle and moment during the weight acceptance phase. ANT = anticipated, UNANT = unanticipated. Bottom row: Statistical parametric mapping (SPM) results for the main condition effects.

**Fig. 3:**
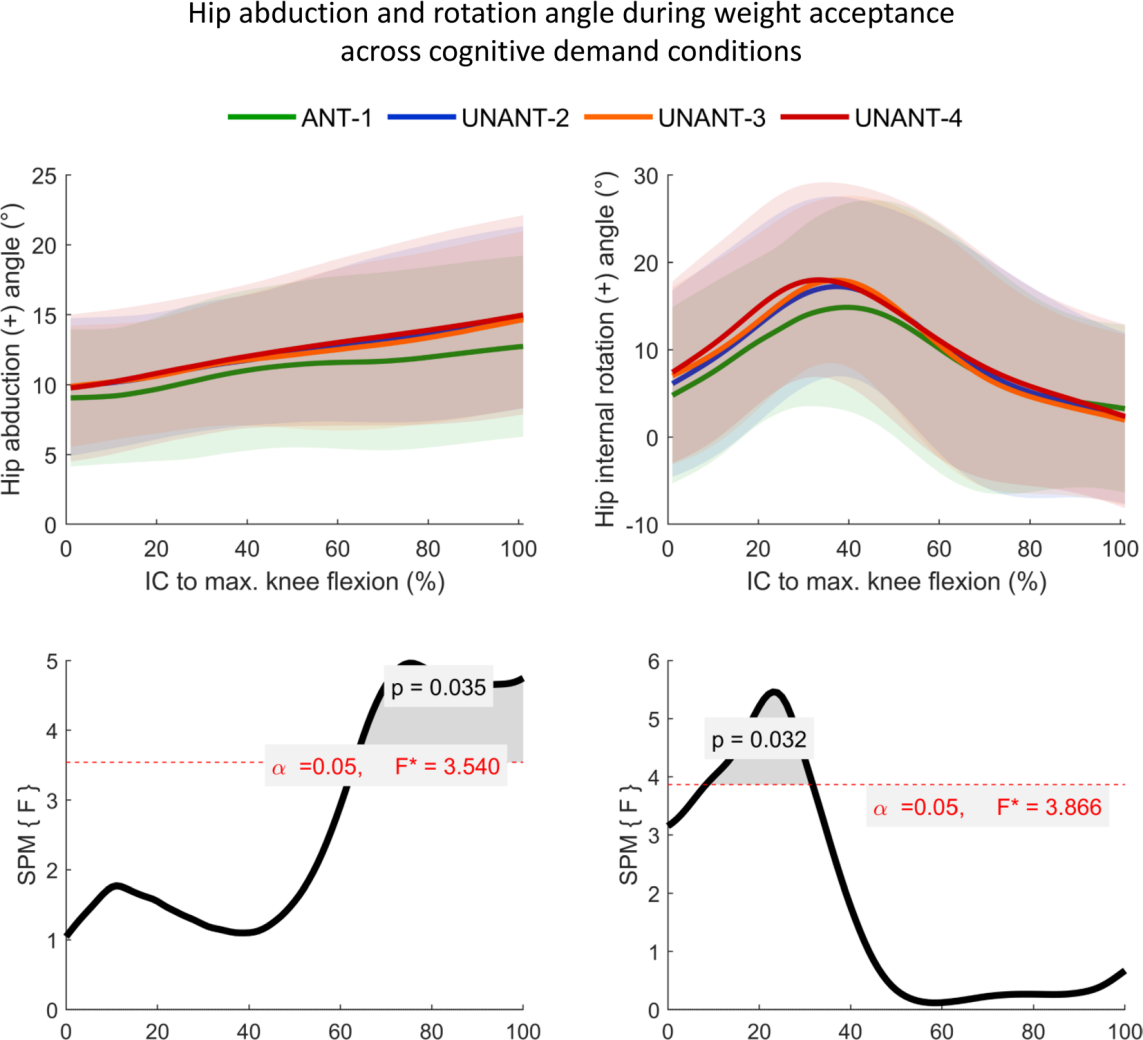
Top row: Means (± SD shaded areas) of hip abduction and rotation angle during the weight acceptance phase. ANT = anticipated, UNANT = unanticipated. Bottom row: Statistical parametric mapping (SPM) results for the main condition effects.

The SPM-analyses revealed no significant main condition effects for the parameters knee abduction moment and knee flexion angle during WA. Significant differences in knee abduction angle were found from 39% − 48% of WA (Fig. 2, *p* = 0.0458, F = 3.975). However, Bonferroni-corrected post-hoc analyses did not reach the level of statistical significance. Regarding hip kinematics, significant differences were found for the hip abduction angle (Figs. 3 and 63% − 100% of WA, *p* = 0.035, F = 3.540) and the hip rotation angle (Figs. 3 and 8% − 32% of WA, *p* = 0.032, F = 3.866), again without reaching significance through post-hoc tests.

## Discussion

In female football, ACL injuries most frequently occur during cognitively demanding match situations—particularly when players execute rapid COD maneuvers in response to an opponent’s action^5,6^. Evidence from previous laboratory-based studies suggests that cognitively demanding unanticipated CODs can modulate biomechanical parameters associated with ACL injuries^13,15^. However, to date, the impact of systematically elevating cognitive demand—by progressively increasing both the number and complexity of available movement options—on these underlying mechanisms remains poorly understood.

In this study, this research gap was addressed by creating complex match-play scenarios in which female athletes executed a 90° COD task in response to a real opponent, while systematically elevating cognitive demand across 4 levels by increasing the number of available movement options. It is noteworthy to add that only CODs to one side were analyzed, while the remaining movement options served to increase the cognitive demands. The secondary aim was to determine whether football-specific expertise influences biomechanical responses to progressively elevated cognitive demands.

Contrary to our expectations, no significant main condition effects were found for peak knee abduction angles, knee flexion angles at IC, or for peak knee abduction moments. However, a trend toward increased peak knee abduction moments were found with increasing cognitive demands. This is in line with findings from a recently published meta-analyses^21^, which compared anticipated and unanticipated CODs in physically active individuals. Although this meta-analysis reported no significant pooled effect of anticipation, more than half of the included studies revealed higher knee abduction moments in unanticipated conditions^21^. For instance, Bill et al. (2022) showed higher knee abduction moments when participants reacted to a movement of a real opponent compared to anticipated trials^18^. In another study, Lee et al. (2013) showed that unanticipated trials elicited by video-animated opponents produced elevated knee abduction moments, but interestingly even higher moments were observed in arrow‐cued COD maneuvers^17^. The authors suggested that ecologically valid match-play stimuli, such as video-based opponents, may prolong the athlete’s ATR by providing earlier visual cues about the intended direction^17^. Conversely, the cognitive demands required to interpret complex visual information and to generate an appropriate motor response are likely to be higher than for simple artificial stimuli^14^.

One possible explanation for the absence of significant main condition effects at the knee joint level in our study may therefore lie in the characteristics of the ATR. Previous studies identified an ATR of around 600 ms to be required to differentiate unanticipated from anticipated CODs at an approach speed of 4.0 m/s^21^. Accordingly, consistency of ATR and approach speed across the conditions was confirmed by good ICCs and no condition effects for all control parameters. However, while ATR_Ball_ remained within the predefined range of 300–600 ms, ATR_Opp_–defined as the time from the initial movement of the opponent’s kicking foot to the participant’s IC on the force plate–was between 700 and 750 ms. Accordingly, the resulting ATR in the respective trials may have varied depending on whether the participants focused on the opponent’s body movement or the ball trajectory to obtain visual cues for the requested movement option. It can therefore be speculated that the prolonged ATR_Opp_ may have diminished condition effects on several biomechanical outcomes, including knee joint mechanics.

No statistically significant main condition effects were found for hip abduction/adduction angles at IC. However, significant main condition effects were observed for hip abduction during WA, and for the internal rotation angle during both IC and WA. Even though post-hoc analyses did not reach the level of statistical significance, visual inspection of the continuous waveforms (Fig. 3) suggests increased hip abduction and internal rotation across all unanticipated conditions compared to ANT-1. These findings are consistent with outcomes from previous studies^18,19,22,30^ and gain additional relevance when considered alongside the findings from video analyses that revealed pronounced hip abduction and internal rotation during ACL injury occurrence^5,6^. Since the hip and knee joint are mechanically linked via the femur, alterations of hip joint kinematics can directly contribute to altered or even detrimental knee mechanics^31^.

In addition, pelvis kinematics were significantly influenced by cognitive demands. In the cognitively most demanding UNANT-4 condition, the pelvis was significantly less laterally tilted towards the new running direction, i.e., with the iliac crest being higher toward the new running direction, compared to the ANT-1 condition. This is in line with a finding from a previous study^32^ which suggests that greater lateral pelvis tilt in anticipated CODs facilitates lateral foot placement, thereby enabling the generation of higher push-off forces, while reducing the need for pronounced hip abduction angles in the cutting leg^32^. This proposed mechanism aligns with our findings of increased hip abduction during WA in unanticipated conditions. Furthermore, the pelvis rotation to the new running direction was significantly reduced in UNANT-4 compared to ANT-1, which indicates delayed reorientation in the most demanding cognitive condition. In this sense, reduced pelvis rotation may increase the need for high hip internal rotation to achieve the desired direction change^33^; a pattern reflected in our results showing a main condition effect on hip rotation during WA. In summary, these findings highlight the role of pelvis kinematics and control for modulating hip and knee joint mechanics during COD performance^31^.

At the trunk level, a significantly more pronounced rotation to the cutting leg was observed in the ANT-1 condition compared to the other cognitive demand conditions. Interestingly, no significant main effects of condition on lateral trunk flexion were found, which is not in line with previous studies^16,17,32^. Given the large mass of the trunk and its influence on the body’s center of mass, lateral trunk flexion is discussed to increase external knee abduction moments by shifting the ground reaction force vector laterally relative to the knee joint^32,33^. In this context, Powers et al. (2010) emphasized the contributing role of proximal segments and joints, especially in the frontal plane, with respect to ACL injuries^31^. With this in mind, the absence of effects on lateral trunk flexion in the current study might help to explain why knee abduction biomechanics also were not affected by increasing cognitive demands. Furthermore, the trunk and hip joint are characterized by a great range of motion, whereas knee frontal plane mechanics are inherently limited. Given this, potential effects of increasing cognitive demands may have primarily emerged at the proximal joints, while subtle changes of knee joint mechanics may have been too small or covered by measurement inaccuracies.

It is important to note that pelvis and trunk rotation were quantified using different reference systems. Pelvis rotation was quantified relative to the global coordinate system, whereas trunk rotation was computed relative to the pelvis. This methodological distinction may explain the seemingly contradictory finding that, in the anticipated condition, the pelvis was more rotated to the new running direction, whereas the trunk was more rotated towards the cutting leg. However, this in line with Byrne et al. (2022), who also reported global pelvis and intersegmental trunk angles^32^. When reporting intersegmental trunk angles, pelvis kinematics should always be reported alongside to allow interpretation of the contribution of pelvis and trunk segments to whole-body biomechanics^32^. Additionally, this information may assist practitioners to decide whether to prioritize pelvis or trunk control or to consider both when developing ACL injury prevention strategies.

In summary, our findings suggest that increasing cognitive demands during COD task performance primarily affect proximal kinematic strategies, particularly at the hip, pelvis, and trunk. As the post-hoc analyses did not reveal significant differences between the 3 unanticipated conditions, our measurement paradigm may be considered only partially successful in differentiating between increasing levels of cognitive demands. However, while these analyses did not reach the level of statistical significance, visual inspection indicated gradual effects on knee abduction moments, pelvis kinematics and hip with increasing cognitive demands. Regarding the pelvis, only the UNANT-4 condition had a significant effect on kinematics, indicating that complex conditions with a high number of movement options are required to affect the movement control of female athletes in a sport-specific scenario.

Based on findings of Lee et al. (2013), it was hypothesized that higher football-specific expertise would reduce the biomechanical effects of increasing cognitive demands^17^. However, no effect of football-specific expertise was found for any of the biomechanical variables examined in this study. A possible explanation is that the groups might have been relatively similar with regard to their expertise in performing unanticipated CODs in team sports. Six of the LE participants played handball (*n* = 3) or rugby (*n* = 3) and may have benefited from their experience in these sports in terms of anticipating and interpreting an opponent’s movements. In this context, the use of artificial turf and football-specific footwear instead of a standard laboratory floor and neutral shoes could favor the investigation of expertise on biomechanical outcomes. Furthermore, player position may have influenced familiarity with and thus execution of COD movements, as the HE group comprised full backs (*n* = 3), centre backs (*n* = 4), central midfielders (*n* = 3), players covering full back and wide midfield (*n* = 2), central forwards (*n* = 2), and a goalkeeper (*n* = 1). According to Dos’Santos et al. (2022), central midfield players perform significantly more CODs with small (20–59°), moderate (60–119°), and large (120–180°) cutting angles during match play compared to players in other positions^34^. Accordingly, future studies might investigate whether playing position moderates the influence of cognitive demands on COD biomechanics.

Although the sample size was based on an a priori power analysis, it was relatively small in light of the multiple comparisons, which is reflected in non-significant post-hoc analyses. Beyond that, three players with previous ACL injuries (> 24 months post-treatment) were involved in this study and may have caused bias due to potential long-term neuromuscular adaptions. Nevertheless, biomechanical data from players with previous ACL injuries and players without injuries were compared but no statistically significant and systematic differences were observed.

Due to the ecological valid setup with a real opponent, the precise ATR of the respective participants—whether based on the ball or the opponent—remains unknown. Future studies might overcome this limitation for instance through eye-tracking. Additionally, a higher temporal variability must be assumed when using ecologically valid compared to artificial stimuli. Furthermore, the setup may have allowed for a certain level of anticipation, as subtle differences in the initiation of the defender’s actions—both within and between the four test conditions—may have been perceived by the participants.

Finally, beyond sport-specific expertise, individual cognitive abilities such as reaction time and processing speed may be crucial for unanticipated COD task performance^35^ and should therefore also be considered in future research.

## Conclusions

In summary, our findings indicate that increased cognitive demands during COD task performance in female football players predominantly influence proximal kinematics at the hip, pelvis and trunk, rather than knee joint mechanics. This supports the relevance of proximal control strategies in injury-related scenarios characterized by elevated cognitive demands. Moreover, the acceptable ICCs for approach speed as well as ATR_Opp_ and ATR_Ball_ indicate that the test setup successfully created a reliable and ecologically valid scenario within a controlled laboratory environment.

Based on our findings, the authors recommend that practitioners and coaches integrate both whole-body control and cognitively demanding decision-making tasks into testing and injury prevention strategies for female football players regardless of their expertise level. With reference to the principle of training specificity, such tasks, including accelerations, quick CODs in response to an opponent and decelerations, could be included into structured warm-up routines or be integrated in neuromuscular training programs.

## Data Availability

The datasets generated and analyzed during the current study are available from the corresponding author on reasonable request.
